# Splenic cysts: Analysis of 16 cases

**Published:** 2016

**Authors:** Hamed Golmohammadzadeh, Ghodratollah Maddah, Yavar Shams Hojjati, Abbas Abdollahi, Hossein Shabahang

**Affiliations:** 1 Endoscopic and Minimally Invasive Surgery Research Center, Mashhad University of Medical Sciences, Mashhad, Iran.; 2 Surgical Oncology Research Center, Mashhad University of Medical Sciences, Mashhad, Iran.

**Keywords:** Splenic cysts, Open splenectomy, Management

## Abstract

**Background:**

Splenic cysts are known as rare clinical encounter. Classifying into primary (true) and secondary cysts (pseudocysts), true cysts contain cellular epithelial lining and subdivided into parasitic and non-parasitic cysts. This study aimed to determine the outcome of treatment in patients with splenic cyst.

**Methods:**

All patients with splenic cyst who had been treated in Department of General Surgery of Ghaem and Omid teaching hospitals over a 24-year period were identified. The medical records of these 16 patients were reviewed.

**Results:**

The study patients included 11 females (68.75%) and 5 males (31.25%) with average age of 39.8 years. Fifteen cases had true cyst including 11 parasitic cysts (hydatid) and only one pseudocyst. 37.5% of the splenic cysts had coexistent cysts in liver, pelvis, omentum and paracolic regions. Nine patients underwent total splenectomy and 5 cases partial splenectomy and 2 remaining cases received conservative medical treatment. The size of the cysts varied from 6 to 25 centimeter with average size of 14.3 centimeter. All patients with hydatid cysts received albendazole postoperative medical treatment with albendazole for 6 months. All patients recovered after treatment.

**Conclusion:**

Open splenectomy whether total or partial is effective and safe in patients with splenec cysts with or without hydatidosis. The outcome of treatment is good without recurrences.

Nowadays, splenic cysts are known as rare clinical condition with 0.07% incidence in general population. According to the presence or absence of cellular epithelial lining, these cysts are classified into primary (true) and secondary (false) cysts. Primary cysts are subdivided into parasitic (60%) and non-parasitic cyst due to their etiology. Nonparasitic cysts are commonly congenital. These cysts present mostly at young age and are located in the upper pole of the spleen (1-3). Parasitic cysts with frequency of 60% are the most common primary cysts, resulting typically from Ecinococus granulosus infection as the third most frequent affected site primarily by this parasite (3). On the other hand, secondary splenic cysts include 75% of nonparasitic types and may spread after blunt abdominal trauma (2). Although many splenic cysts are asymptomatic, but dull pain in upper abdominal area due to mass effect may be the presenting feature of these patients (4). 

## Case presentation

Between 1990 and 2014, all patients who were diagnosed with splenic cyst and being treated at Department of General Surgery of Ghae0m and Omid teaching Hospitals were identified.

The demographic data, chief complaint, final diagnosis based on pathology result, coincident lesions, treatment and cyst size based on computed-tomographic scan were obtained from hospital records. Data were analyzed by statistical software SPSS Version 11.5. Over a 24-year period, 16 patients were diagnosed to have splenic cyst and were treated in our institution. Demographic, surgical and pathologic data are shown in table 1. 

**Table 1:** Demographic, surgical and pathologic data of patients. Research Center of Mashhad University of Medical Sciences, Mashhad, Iran

**sex**	**Age(y)**	**Chief complaint**	**Final diagnosis**	**Coincident lesions**	**Treatment strategy**	**Size of cyst** **(cm)**
**F**	49	Chronic pain due to abdominal trauma	Splenic pseudocyst	-	Total splenectomy	5*7
**F**	19	Abdominal pain started 4 m ago	Epithelial cyst of spleen	-	Total splenectomy	11*12
**F**	16	Abdominal pain started 15 days ago	Epithelial cyst of spleen	-	Total splenectomy	19*21
**F**	21	Abdominal pain	Epithelial cyst of spleen	-	Total splenectomy	15
**M**	25	Abdominal pain & vomiting started 2 m ago	Hydatid cyst of spleen	Liver hydatid cyst	Total splenectomy capitophage of liver cyst	7.5*10
**F**	48	Lumbar pain started 5 m ago	Hydatid cyst of spleen	Left para colic cyst(8cm) Right pelvic cyst(7 cm) Retro bladder and antrorectal cyst(10 cm)	Conservative medical therapy	10
**M**	60	Hypochondriac mass started 2 m ago	Hydatic cyst of spleen	Severe adhesion to pancreas and diaphragm	Total splenectomy distal pancreatectomy repairing the abdominal surface of diaphragm	22
**M**	37	Abdominal pain started 6 m ago	Hydatic cyst of spleen	-	Partial splenectomy	8*17
**M**	33	Abdominal pain started 1 w ago	Hydatic cyst of spleen	Torsion of omentum intra-abdominal bleeding	Partial splenectomy & removed torsion part of omentum	11*12
**F**	16	Abdominal pain started 2 y ago	Hydatic cyst of spleen	Multiple liver cysts	Partial splenectomy	1-6
**F**	37	Abdominal pain with nausea and vomiting started 6 m ago	Hydatic cyst of spleen	Cysts in left and right lobe of liver	Partial splenectomy drainage of liver cysts	9*12
**F**	72	Abdominal pain started 2 m ago	Hydatic cyst of spleen	-	Drainage of 4 liter fluid of cyst then partial resection of cyst’s wall without splenectomy	22
**M**	81	Abdominal pain started 2 w ago	Hydatic cyst of spleen	Several cysts in omentum Pelvic and para colic cyst Liver cyst	Conservative medical therapy	14
**F**	42	Abdominal mass & splenomegaly from 8 y ago Abdominal pain from 10 d ago	Hydatic cyst of spleen and liver	Cyst of omentum Multiple liver cysts Total splenectomy capitophage of liver cysts resection of omentum cyst		25
**F**	44	Abdominal pain due to cholelithiasis Incidental finding of spleen cyst	Epithelial cyst of spleen	cholelithiasis	Cholecystectomy Laparascopic splenectomy	6
**F**	37	Abdominal pain from 1 m ago	Hydatic cyst of spleen	-	Total splenectomy	13*18

The average age of our participants was 39.8(16-81). The sex distribution was 68.75% females (n=11) and 31.25% males (n=5).93.75% of cases had primary or true cyst (n=15), including hydatid cyst in 11 cases. (73.4% of true cysts and 68.75% in overall cases) and epithelial or non-parasitic cysts in 4 cases (26.66% of true cysts and 25% of overall cases). Only one patient had a pseudocyst (6.25%). Dull abdominal pain was reported in 87.5%. Only one patient suffered from abdominal mass. Incidental diagnosis by imaging evidence of splenic cyst was found in only one patient. Total splenectomy was performed in 56.25 % (n=9), and partial splenectomy was employed for 31.25% (n=5) While 12.5% of cases (n=2) underwent conservative medical treatment. The average size of the cysts was 14.3 centimeter (maximum size=25, minimum size=6 cm). 37.5% of splenic cyst had coexistent involvement of formation of cyst in other organs, including liver cyst in 5 cases, cyst of omentum in 2 cases and pelvic and para colic cyst in 2 cases. Only one case of cholelithiasis and one case of omentum-torsion were reported.

## Discussion

Splenic cysts are rare condition, particularly non-parasitic ones that accounted for about 30-40% of overall splenic cysts (5). Among the parasitic ones, being the most common type of primary splenic cysts, cyst formation due to Ecinococus granulosus infection with incident of 60 percentage is the most frequent etiology. Pseudocyst as a result of hematoma secondary to blunt abdominal trauma is an uncommon finding. (3). Although many of splenic cysts are asymptomatic, upper abdomen dull pain may be presented due to mass effect of the enlarged cyst or strain of the splenic capsule (4). 

Only one asymptomatic case was reported in this trial, while most of cases were symptomatic and abdominal pain was the most common chief complaint (75 percent of our studied population). The usefulness of unenhanced computed tomography scan imaging in the diagnosis of splenic cyst especially splenic hydatid disease is well-established. However ultrasonography also yields comparable diagnostic ability, although the findings are not specific (6).

**Figure 1:**
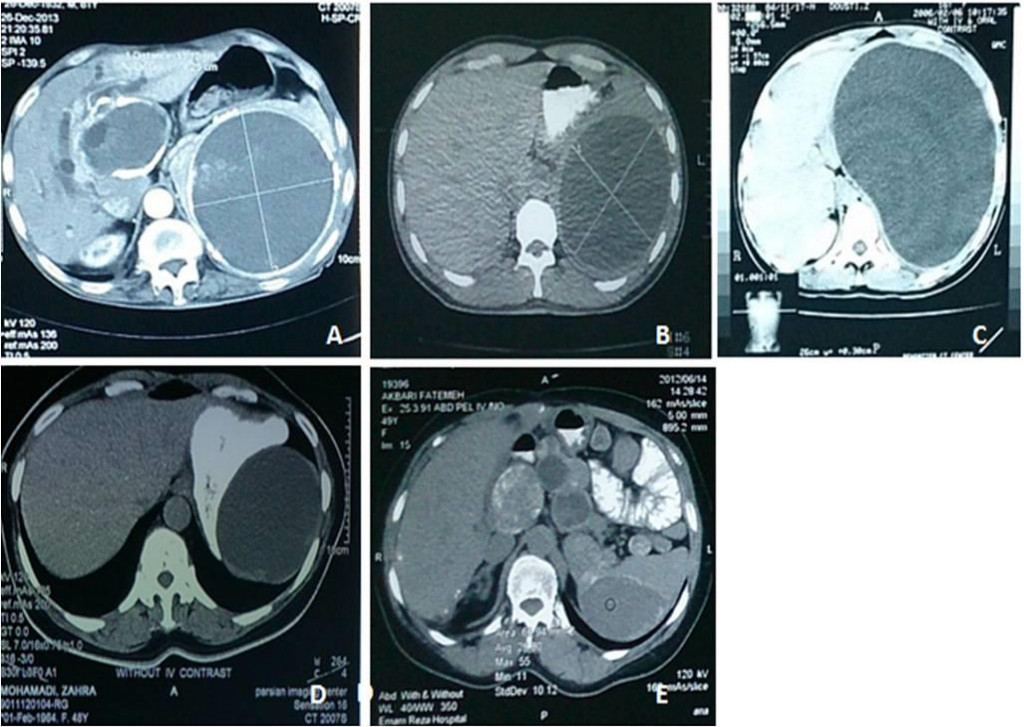
remarkable CT-scan findings in our trial;

CT-scan findings of parasitic cysts include well-defined lesions, homogeneous fluid content with attenuation values same as water, no contrast-enhancement, wall calcifications, water lily sign and mass effect (7). We performed CT-scan imaging as the diagnostic method for all cases. Figure 1 presents some remarkable CT-scan findings of our patients. A different management is required for parasitic and non-parasitic cysts due to their different pathogenesis (3). Size of cyst and related symptoms are major factors which affected treatment decision (8). While small asymptomatic cysts(less than 5 cm in diameter) especially non-parasitic ones (traumatic secondary cysts) are best followed conservatively with serial imaging due to their spontaneous resolving, larger cysts (more than 5 cm in diameter) and symptomatic cysts must be treated surgically due to their susceptibility of hemorrhage, rupture and infection (peritonitis and abscess formation) (9-13).

In our study, surgical management was undertaken in 87.5%, while 12.5% of cases (n=2) underwent conservative medical treatment. Although splenic involvement is the third common site of hydatid disease but the diagnosis often is made incidental because of the asymptomatic nature of splenic hydatid cysts (14). Nonetheless, our hydatic splenic cysts presented with a spectrum of clinical symptoms including abdominal pain (the most common presentation), lumbar pain, nausea and vomiting. Surgical management options of splenic cyst include percutaneous drainage, total splenectomy and partial splenectomy. However, application of this method is controversial disease recurrences and high chance of adhesion and inflammation of the spleen, nevertheless this method can be used as preoperative management for decreasing cyst size. Generally spleen-preserving surgery methods must be the leading aim of management of splenic cysts, besides under especial situations like polycystic cases, enormous cyst size, hilar cyst, cyst with fully parenchymal coverage, uncontrollable massive bleeding and inaccessible cysts, the patient is candidate for total splenectomy (9, 10, 15). Conserving 25% of the spleen’s parenchyma through partial splenectomy regularly provides the sufficient immunologic function for the patient (10, 16). Other management strategies include marsupialization, fenestration and laparoscopic unroofing. Common disadvantages of these methods are high recurrence rate and bleeding chance (9, 15). Our experience indicates that both partial and total splenectomy are appropriate method of treatment for splenic cyst which are associated with low rate of recurrences and postoperative complications. Patients with splenic hydatid cysts received albendazole 10 to 15 mg/kg per day (with maximum dose of 800 mg) as post-operative medical treatment for 6 months.

A. 81 year-old male with gallstone (diameter of 11 mm) and multi-cystic lesions with diffuse calcification in liver (maximum diameter of 85 mm) and spleen (maximum diameter of 143 mm).

B. 33 year-old male with non-enhanced hypodense lesion (110*124 mm) suggestive for hydatid splenic cyst.

C. 16 year-old female with huge well-defined cystic lesion with diameter of 200mm and its mass effect.

D. 49 year-old female with splenic pseudocyst (50*70 mm)

E. 48 year-old female with left paracolic cyst (80 mm), Right pelvic cyst (70 mm), retro bladder and antrorectal cyst (100 mm) and splenic cyst (100 mm).

## Conclusion:

Open splenectomy whether total or partial is effective and safe in our limited experience. We strongly suggest surgical management as main treatment of symptomatic and parasitic splenic cysts instead of a follow-up with medical treatment. And also, it seems that there is no need for follow-up after surgery. Further studies are required to determine the best management of asymptomatic cysts in larger population. Generally spleen-preserving surgery, whether laparoscopic or open surgery, must be the leading aim of the management of nonparasitic primary splenic cysts.

## Notes

**Funding:** This work was supported by the Vice Chancellery for Research of Mashhad University of Medical Sciences, Mashhad, Iran, and performed in the Endoscopic and Minimally Invasive Surgery Research Center.

## Citation:

Golmohammadzadeh H, Maddah G, Shams Hojjati Y, et al. Splenic cysts: Analysis of 16 cases. Caspian J Intern Med 2016; 7(3): 217-221.
